# The Metalloprotease-Disintegrin ADAM8 Alters the Tumor Suppressor miR-181a-5p Expression Profile in Glioblastoma Thereby Contributing to Its Aggressiveness

**DOI:** 10.3389/fonc.2022.826273

**Published:** 2022-03-15

**Authors:** Agnes Schäfer, Lara Evers, Lara Meier, Uwe Schlomann, Miriam H. A. Bopp, Gian-Luca Dreizner, Olivia Lassmann, Aaron Ben Bacha, Andreea-Cristina Benescu, Mirza Pojskic, Christian Preußer, Elke Pogge von Strandmann, Barbara Carl, Christopher Nimsky, Jörg W. Bartsch

**Affiliations:** ^1^ Department of Neurosurgery, Philipps University Marburg, Marburg, Germany; ^2^ Marburg Center for Mind, Brain and Behavior (MCMBB), Marburg, Germany; ^3^ Core Facility Extracellular Vesicles, Philipps University of Marburg – Medical Faculty, Marburg, Germany

**Keywords:** glioblastoma, tumor microenvironment, extracellular vesicles, miRNA, MR spectroscopy, ADAM8, miR-181a-5p, MMP9

## Abstract

Glioblastoma (GBM) as the most common and aggressive brain tumor is characterized by genetic heterogeneity, invasiveness, radio-/chemoresistance, and occurrence of GBM stem-like cells. The metalloprotease-disintegrin ADAM8 is highly expressed in GBM tumor and immune cells and correlates with poor survival. In GBM, ADAM8 affects intracellular kinase signaling and increases expression levels of osteopontin/SPP1 and matrix metalloproteinase 9 (MMP9) by an unknown mechanism. Here we explored whether microRNA (miRNA) expression levels could be regulators of MMP9 expression in GBM cells expressing ADAM8. Initially, we identified several miRNAs as dysregulated in ADAM8-deficient U87 GBM cells. Among these, the tumor suppressor miR-181a-5p was significantly upregulated in ADAM8 knockout clones. By inhibiting kinase signaling, we found that ADAM8 downregulates expression of miR-181a-5p *via* activation of signal transducer and activator of transcription 3 (STAT3) and mitogen-activated protein kinase (MAPK) signaling suggesting an ADAM8-dependent silencing of miR-181a-5p. In turn, mimic miR-181a-5p transfection caused decreased cell proliferation and lower MMP9 expression in GBM cells. Furthermore, miR-181a-5p was detected in GBM cell-derived extracellular vesicles (EVs) as well as patient serum-derived EVs. We identified miR-181a-5p downregulating MMP9 expression *via* targeting the MAPK pathway. Analysis of patient tissue samples (n=22) revealed that in GBM, miR-181a-5p is strongly downregulated compared to *ADAM8* and *MMP9* mRNA expression, even in localized tumor areas. Taken together, we provide evidence for a functional axis involving ADAM8/miR-181a-5p/MAPK/MMP9 in GBM tumor cells.

## Introduction

Glioblastoma multiforme (GBM) is the most common malignant primary brain tumor in adults. Despite a standard multimodal therapeutic strategy combining maximum safe surgical resection and radio-/chemotherapy with temozolomide, the median survival remains low between 12 and 15 months (1). To improve the poor prognosis of GBM patients it is crucial to identify new therapeutic targets and their underlying dysregulated signaling pathways.

GBM is characterized as a highly invasive, heterogeneously composed, and rapidly growing tumor (2). At the molecular level, a disintegrin and metalloproteinases (ADAMs) mediate tumor cell adhesion and migration as well as intracellular signaling (3). One such proteolytically active family member is the metalloproteinase-disintegrin 8 (ADAM8), strongly associated with tumor aggressiveness, progression, and reduced survival in various cancers including breast cancer, pancreatic ductal adenocarcinoma (PDAC), and GBM (4–7). ADAM8, in particular, the cytoplasmic domain (CD) and the disintegrin/cysteine-rich domain (DC) can activate central signaling pathways in carcinogenesis. First, ADAM8 activates the mitogen-activated protein kinase (MAPK) signaling cascade, epidermal growth factor receptor (EGFR) independently (8, 9). Second, ADAM8 mediates angiogenesis by inducing the expression of osteopontin (*SPP1*) *via* STAT3 signaling (10). Moreover, ADAM8 interacts with integrin ß1 (ITGB1) and thereby activates its downstream targets focal adhesion kinase (FAK), and the PI3K/AKT pathway (9, 11). Interestingly, ADAM8 dependent activation of the MAPK pathway as well the PI3K/AKT pathway enhanced temozolomide-chemoresistance in GBM cell lines (12). Considering these diverse functions of ADAM8 in intracellular signaling, we and others hypothesized that ADAM8 mediates these functions through the regulation of microRNAs and indeed, initial evidence came from a study in MDA-MB-231 breast cancer cells showing that ADAM8 regulates expression levels of miR-720 (13).

MicroRNAs (miRNAs) are small non-coding RNA molecules that regulate protein expression on a post-transcriptional level by binding and thereby silencing their target messenger RNAs (mRNAs) (14). In most cases, miRNAs lead to translational repression or even degradation of their specific target mRNAs (15). Therefore, dysregulated miRNA expression profiles alter many critical pathways related to cancer progression (16). Consequently, in GBM, a large number of miRNAs are reported to be dysregulated (17, 18). In GBM, miR-181a-5p is downregulated and functions as a tumor suppressor miRNA that inhibits the translation of oncogenic proteins that are linked to tumor progression such as osteopontin (*SPP1*) (19–21). This type of sialoprotein is highly expressed in GBM and plays a key role in tumor-tumor microenvironment communication by attracting macrophages and mediating their immune response (22). Furthermore, miR-181a-5p regulates cell apoptosis and cell colony formation by targeting B-cell lymphoma 2 (BCL-2), so that high expression levels of miR-181a-5p can induce radiosensitivity of U87 GBM cells (23, 24). In addition, miR-181a-5p contains inhibitory binding sites to members of the MAPK family and its downstream targets, namely mitogen-activated protein kinase kinase 1 (MEK1), cAMP response element-binding protein 1 (CREB-1), and extracellular signal-regulated kinase 2 (ERK2) (25, 26). Given an important functional role in GBM, the signaling pathways regulating miR-181a-5p itself, however, remain unclear.

Matrix metalloproteinase 9 (MMP9), a zinc-dependent endopeptidase, plays a central role in the process of tumor cell migration, infiltration, and metastasis (27). Matrix metalloproteinases degrade extracellular matrix molecules and basement membrane components and thereby contribute to glioma progression (28). Consequently, MMP9 is upregulated in GBM compared to its expression in the normal brain parenchyma (29). Gliomas that display high MMP9 levels are associated with an aggressive course and are linked to reduced survival (30). Previous studies demonstrated that MMP9 expression can be elevated *via* MAPK-signaling (31, 32). ADAM8 and MMP9 levels are correlated in GBM tissue samples as well breast cancer-derived brain metastasis (8, 33). Whether MMP9 can be directly targeted by miR-181a-5p or indirectly *via* miR-181a-5p induced downregulation of the MAPK pathway has not been explored yet.

Cancer invasion is closely associated with the interaction of infiltrating tumor cells and the tumor microenvironment (TME) (34). As a means of communication, extracellular vesicles (EVs) are secreted by tumor cells as well as by cells of the TME. Their cargo contains lipids and proteins as well as nucleic acids including miRNAs (35). Because EVs modulate tumor growth, immune-escape, and tumor cell niche formation, they function as central regulators of the TME (34).

In the current work, we explored the mechanism by which ADAM8 modulates intracellular and extracellular signaling through the regulation of miR-181a-5p expression and uncovered *MMP9* as a miR-181a-5p dependent target gene in GBM.

## Material and Methods

### Patient Specimens

In accordance with the local ethics committee (Philipps University Marburg, medical faculty, file number 185/11), tumor tissue samples of GBM patients were obtained during surgical resection and serum specimens were collected one to three days prior and three to five days after surgical resection. Each patient gave written informed consent before resection. Tissue samples were shock frozen in liquid nitrogen and then stored at -80°C. Serum samples were centrifuged at 2,000 g for 10 min prior to storage at -80°C. All included tissue and serum samples were from primary, isocitrate dehydrogenase (IDH) wild-type GBM tumors, further patient information and histopathological characteristics are summarized in **Table 1**. In three cases, we analyzed the expression of miR-181a-5p in serum-derived EVs at the time of initial manifestation and tumor recurrence (Patient 9, 23, 24 in **Table 1**).

**Table 1:** Clinical data on patient included tumor tissue samples showed isocitrate dehydrogenase (IDH) wild-type expression.

Number	Age at diagnosis (years)	Sex	Tumor localization	Type of resection	MGMT promoter meth ylation	EGFR vIII	Ki67-Li	Survival (days)
1	71	m	Septum pellucidum	Subtotal	Methylated	–	Up to 30%	114
2	65	m	Left parietal lobe	Subtotal	Not methylated	+	Up to 40%	119
3	77	w	Right frontal lobe	Subtotal	Methylated	–	Up to 20%	79
4	75	m	Right temporal and parietal lobe	Subtotal	Methylated	–	10%	476
5	63	w	Left frontal lobe	Subtotal	Methylated	–	Up to 30%	76
6	87	m	Right parietal and occipital lobe	Gross Total	Methylated	++	5%	135
7	78	w	Butterfly glioma, predominantly right frontal lobe	Subtotal	Not methylated	unknown	20%	63
8	66	m	Left frontal lobe	Subtotal	Methylated	–	25%	49
9*	66	m	Right occipital lobe	Gross total	Not methylated	+	20%	336
10	65	w	Left temporal lobe	Subtotal	Not methylated	–	Up to 15%	84
11	70	w	Left frontal lobe	Subtotal	Methylated	+	Up to 25%	278
12	61	m	Right temporal lobe	Gross total	Methylated	–	30%	626
13	64	w	Right frontal and temporal lobe	Gross total	Methylated	–	20%	930
14	65	m	Left temporal lobe	Subtotal	Methylated	–	30%	579
15	66	m	Right temporal lobe and right Insula	Subtotal	Methylated	–	Up to 20%	126
16	61	m	Left temporal lobe	Gross total	Not methylated	–	50%	398
17	57	w	Right frontal lobe	Gross total	Weakly methylated	+	20%	410
18	62	m	Right temporal and parietal lobe	Gross total	Not methylated	–	Up to 20%	457
19	56	m	Left temporal lobe	Gross total	Methylated	–	20%	578
20	69	m	Right parietal and occipital lobe	Gross total	Weakly methylated	–	Up to 50%	388
21	61	m	Right frontal lobe	Gross total	Weakly methylated	–	30%	94
22	76	m	Right frontal lobe	Gross total	Not methylated	–	30%	225
23*	76	f	Left parietal	Gross total	Methylated	–	20%	unknown
24*	54	m	Right frontal lobe	Gross total	Methylated	–	30%	450
25	74	f	Left parietal lobe	Gross total	Methylated	–	40%	unknown

Only initial manifested primary glioblastomas were included. Here, we show further parameters regarding the patient cohort including age at diagnosis, sex, survival in days, and type of surgical resection (gross total or subtotal). Furthermore, histopathological data such as methylation status of the O^6^-methylguanine-DNA-methyltransferase (MGMT), Ki67-Labelling index (Ki67-Li), and expression of epidermal growth factor variant III (EGFRvIII) are presented here. Patients’ 1 to 22 tissue samples were analyzed for miR-181a-5p, *ADAM8*, and *MMP9* mRNA expression (**Figures 5A–D**), matched samples (initial and recurrence GBM) from patients 9, 23, and 24 (*) were used for serum-EV separation and analysis (**Figures 5H–J**) and patient 25 was used for the analysis via MR-spectroscopy (**Figures 5E–G**).

### Cell Culture

Established GBM cell lines U87 and U251 were purchased from the American Type Culture Collection (ATCC) and cell lines G112 and G28 were obtained from the Westphal Lab (UKE Hamburg). All GBM cell lines were cultivated in Dulbecco’s modified Eagle’s medium (DMEM) high glucose (4.5 g/L) phenol red (Capricorn Scientific, Germany), supplemented with 10% fetal calf serum (FCS, S0615, Sigma, Germany), 1% penicillin/streptomycin (2321115, Gibco, US), 1% sodium pyruvate (NPY-B, Capricorn Scientific, Germany) and 1% non-essential amino acids (11140050, Gibco, US). Primary GBM cell lines and primary glioblastoma stem-like cells (GSCs) were obtained during surgical resection. The isolation and preparation process of GSCs and primary differentiated patient-derived GBM tumor cells were each described previously by our group (12, 36). GSC lines 2017/151, 2017/74, and 2016/240 were cultivated in DMEM/F12 (DMEM-12-A, Capricorn Scientific, Germany) and supplemented with 2% B27 supplement (117504044, Gibco, US), 1% amphotericin (152290026, Gibco, US), 0.5% HEPES (H0887, Sigma, Taufkirchen, Germany) and 0.1% Gentamycin (A2712, Biochrom, Germany). Moreover, a final concentration of 0.02 ng/µL EGF (100-18B, Peprotech, Germany) and bFGF (315-09, Peprotech, Germany) was added, and GSCs were cultivated in non-cell-culture-treated petri dishes. Primary differentiated GBM cell lines GBM98, GBM42, and GBM29 were cultivated in DMEM high glucose (4.5 g/L) without phenol red (Capricorn Scientific, Germany) supplemented with 10% FCS (S0615, Sigma, Germany), 1% penicillin/streptomycin (2321115, Gibco, US), 1 mM sodium pyruvate (NPY-B, Capricorn Scientific, Germany), 1% L-glutamine (200 mM) (25030-024, Gibco, US) and 1% non-essential amino acids (11140050, Gibco, US). All cell lines were cultured in a humidified atmosphere at 37°C under 5% CO_2_.

### Generation of Stable U87 CRISPR/Cas9 ADAM8 KO (KO) Clones

U87 cells were transfected with two different gRNAs using the CRISPR/Cas9 knockout/knockin kit from OriGene (# KN213386) as described previously (37). Cell clones were selected by treatment with antibiotics (1 mg/ml puromycin). The ADAM8 knockout was confirmed through RT-qPCR, western blot, and ELISA analysis. U87 wild-type cells were used as control cells.

### Transient Transfection to Induce an ADAM8 Rescue in U87 ADAM8 KO Cells

To rescue ADAM8 in U87 ADAM8 KO clones, cells were seeded in 6-well-plates at a density of 500,000 cells in 2 ml. After 24 h, the transfection was performed with either ADAM8 lacking the cytoplasmatic domain or the full-length ADAM8 using LTX Lipofectamine (Invitrogen) according to the manufacturer’s instructions. Cells were harvested and analyzed by RT-qPCR and western blot after 48 h of transfection.

### MiR-181a-5p Mimic Transfection

To transiently overexpress miR-181a-5p, U87 cells were seeded in 6-well-plates at a density of 400,000 cells in 2 ml and were transfected with 0.01 µM miR-181a-5p mimic (miScript, Qiagen) after 24 h. 0.01 µM ON-TARGET *plus* non-targeting Control Pool (Dharmacon, US) was used as control RNA. Transfection was performed utilizing Lipofectamine RNAimax (Invitrogen, UK) according to the manufacturer’s instructions. After 24 h, the transfection was repeated. Transfected cells and their controls were harvested 48 h after the second transfection. To evaluate the success of transfection, miRNA expression was analyzed by RT-qPCR.

### Inhibitors

Batimastat was used as a broad-spectrum MMP-inhibitor and was purchased from Tocris (Biotechne, Wiesbaden, Germany). As a specific ADAM8-inhibitor, BK-1361 (Peptide 2.0) was utilized and described by our group previously (9). WP´066 (Sigma Aldrich, US) was used as a JAK2/STAT3 inhibitor. Cells were seeded in a 6-well-format (500,000 cells in 2 ml) and harvested 16 h after treatment with inhibitors. The concentrations used are indicated in the graphs.

### Separation of Extracellular Vesicles (EVs)

EVs were separated from cellular supernatants and GBM patients’ serum samples *via* sequential ultracentrifugation. Cells were incubated with 30 ml DMEM supplemented with 1% L-glutamine (200 mM), 1% penicillin/streptomycin, 1 mM sodium pyruvate solution, and 1% nonessential amino acids for 48 h. Prior to EV separation, serum samples were diluted 1:3 with HBSS (Gibco™, Life Technologies, US) (500 µl serum diluted with 1 ml HBSS). The conditioned medium and the diluted serum sample were centrifuged first at 2,000 g for 10 min at RT and then at 10,000 g for 60 min at 4°C. After a subsequent filtration (0.2 µm filter), EVs were pelleted *via* high-speed centrifugation at 100,000 g for 90 min at 4°C using an Optima XPN-80 ultracentrifuge (Beckman Coulter, Germany). Next, the EV pellet was washed with HBSS at 100,000 g for 90 min at 4°C using the Optima MAX-XP (Beckman Coulter, Germany) ultracentrifuge with a TLA-55 fixed angle rotor. EVs were resuspended in 50 µl HBSS and stored at -80°C until further use. A 5 µl aliquot was sent to the FACS Core Facility, Marburg, for determining the size and concentration of the particles by usage of nano-flow cytometry (NanoFCM Co. Ltd., Nottingham, UK).

### Real-Time Quantitative Polymerase Chain Reaction (qPCR)

Total RNA with an enriched fraction of miRNAs from tumor tissue samples and cellular pellets was isolated using the miRNeasy Tissue/Cells Advanced Mini Kit (217684, Qiagen, Germany) according to the manufacturer’s instructions. To quantify the miRNA expression in cells, miRCURY LNA RT Kit (Cat. Number 339340, Qiagen, Germany) and miRCURY LNA SYBR^®^ Green PCR Kit (Cat. Number 339345, Qiagen, Germany) were used according to manufacturer’s instructions. YP00203_U6 snRNA miRCURY LNA PCR Assay (YP00203907, Qiagen, Germany) and miRCURY miRNA Assay hsa-181a-5p (YP00206081, Qiagen, Germany) was used for the quantification of relative miR-181a-5p expression. In the case of tissue samples (**Figure 5**), miScript II RT Kit (218161, Qiagen, Germany) and miScript SYBR Green PCR Kit (218073, Qiagen, Germany) were used according to the manufacturer’s protocols. Here, Hs_RNU6-2_11 miScript Primer Assay (MS00033740, Qiagen, Germany) and Hs_miR-181a_2 miScript Primer Assay (MS00008827, Qiagen, Germany) were used. To assess gene expression on an mRNA level, RNA was reverse transcribed with RNA to cDNA EcoDry™ Premix (Takara Bio. Inc.). Quantitative real-time PCR was performed with iTaq™ Universal SYBR Green Supermix (Bio-rad Laboratories GmbH, US). QuantiTect Primer Assay (Qiagen) or forward and reverse primer were used in a total reaction volume of 20 µl. XS13 was used as a housekeeping gene. All PCR experiments were performed on the Applied Biosystems StepOnePlus Real-time PCR system (Thermo Fisher Scientific, US). Relative gene expression was calculated utilizing either the 2^-ΔCt^- or the 2^-ΔΔCt^ -method as indicated.

### MiRNA PCR Array – Human Finder

A pathway-focused miRNA PCR Array/Human Finder (331221 miScript, MIHS-001ZC, Qiagen, Germany) was conducted according to the manufacturer’s instructions. Data analysis was performed with the online miScript miRNA Data Analysis program from Qiagen using the 2^-ΔΔCt^-method. Results are presented in a heatmap.

### Protein Extraction and Western Blot Analysis

Cells were washed with PBS (Sigma-Aldrich, US) and detached by cell scraping. Whole cell lysates were homogenized by an incubation for 30 min in RIPA buffer (50 mM HEPES pH 7.4; 150 mM NaCl; 1% (v/v) NP-40; 0.5% (w/v) Natriumdeoxycholate; 0.1% (w/v) SDS; 10 mM Phenantrolin; 10 mM EDTA; Pierce™ Protease Inhibitor Mini Tablets, EDTA-free, Thermo Fisher Scientific; Pierce™ Phosphatase Inhibitor Mini Tablets, Thermo Fisher Scientific). Protein samples or EVs in a concentration of 1.5x10^9^ particles were prepared in 5x Laemmli buffer [60 mM Tris-HCl pH 6.8; 2% (w/v) SDS; 10% (w/v) Glycerol; 5% (v/v) ß-Mercaptoethanol; 0.01% (w/v) Bromophenol-Blue] and 10x NuPAGE™ sample reducing reagent (Thermo Fisher Scientific, US) and denatured at 95°C for 5 min before SDS PAGE. For this, a 10% SDS polyacrylamide gel was used. Separated proteins were transferred on nitrocellulose membranes (A29591442, GE Healthcare Life science, Germany) followed by blocking in 5% (w/v) milk powder (MP) in TBST (50 mM Tris, pH 7.5; 150 mM NaCl; 0.1% (w/v) Tween-20) for 1 h. The detection of proteins was performed utilizing the following primary antibodies diluted as indicated in 5% MP in TBST: anti-ADAM8 (PA5-47047, Thermo Fisher Scientific, 1:1000), anti-MMP9 (IM09L, Calbiochem, 1:1,000), anti- β-Tubulin (NB600-936, Novus Biological, 1:2,000) anti-EGFR (4267, Cell Signaling, 1:1,000), anti-pEGFR (3777, Cell Signaling, 1:1,000), anti-MAPK (4696, Cell Signaling, 1:2,000), anti-pMAPK (4370, Cell Signaling, 1:2000), anti-CALNEXIN (2679, Cell Signaling, 1:1,000), anti-FLOTILLIN-1 (PA5-18053, Thermo Scientific, 1:2,000) anti-CD81 (sc166029, Santa Cruz, 1:500), anti-STAT3 (ab68153, Abcam, 1:5,000), anti-pSTAT3 (ab76315, Abcam, 1:5,000), anti-CREB-1 (H74) (sc-25785, Santa Cruz, 1:500 in 5% MP) and anti-pCREB-1(Ser133) (4276, Cell Signaling, 1:1000 in 5% BSA in TBST). Nitrocellulose membranes were incubated with primary antibodies at 4°C overnight. After washing three times with TBST, membranes were incubated with horseradish peroxidase (HRP) conjugated antibodies (Abcam, 1:5,000) for 1 h followed by a next washing step. Chemiluminescence detection was performed by adding Western Bright Sirius substrate (Advansta, US) and using the ChemiDoc MP Imaging System (Bio-rad Laboratories GmbH, US). Western blots were quantified using Image J (NIH, Maryland).

### Enzyme-Linked Immunosorbent Assay (ELISA)

Soluble ADAM8 (DY1031, R&D Systems, UK) and soluble MMP9 (DY911, R&D Systems, UK) from cell culture supernatants were determined by Sandwich-ELISA method with DuoSet ELISA Kits. All ELISA experiments were performed according to the manufacturer’s instructions.

### Proliferation Assay

The proliferation and survival effects on U87 cells were determined using CellTiter-Glo 3D cell viability assay (G7571, Promega, Germany). Cells were seeded in triplicates on a 96-well plate. After 24 h, miR-181a-5p mimic was transfected according to section 2.5. After 48 h, 50 µl of CellTiter-Glo 3D Reagent was added to each well and mixed while shaking for 15 min. After an additional 15 min without shaking avoiding light, Luminescence was measured with a Microplate Reader luminometer (FLUOstar OPTIMA Microplate Reader, BMG Labtech, Germany).

### Spectroscopy

A T2-weighted magnetic resonance (MR) tomography together with 1H-MR spectroscopy was performed on a 3T MR System (Trio, Siemens, Erlangen, Germany) for in detail analyses of tumor heterogeneity in patient 25. Thereby, a navigated extraction of tissue samples by co-registration of MR data and integration into the neuronavigation system (Curve Ceiling-Mounted, Brainlab, Munich, Germany) was enabled.

### Statistical Analysis

Student’s t-tests were applied for statistical analysis. For multiple comparisons, two-way ANOVA tests were used. A Wilcoxon-signed rank test and Pearson correlation were performed to determine differences or correlation in gene expression. Results were considered as not significant (ns) (p > 0.05), significant (*) (p < 0.05), highly significant (**) (p < 0.01), or very highly significant (***) (p < 0.001). Data from multiple replicates are presented as mean ± SD and statistical analyses were performed with GraphPad Prism (version 9.1.0) and Microsoft Excel.

## Results

### ADAM8 Regulates Expression Levels of miR-181a-5p in GBM Cells

**Figure 1:**
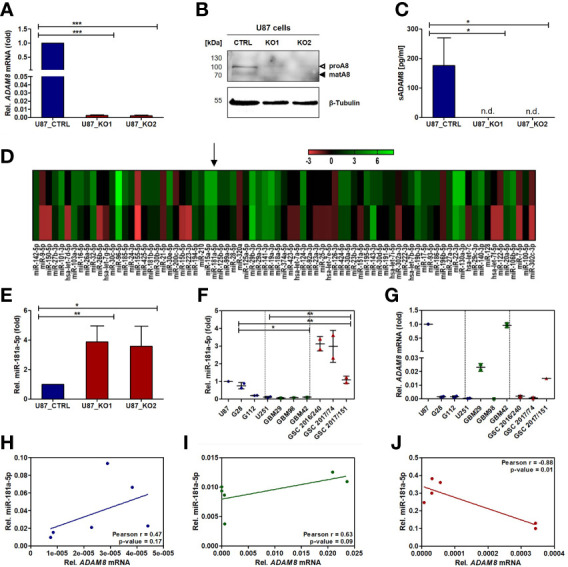
Screening of two representative CRISPR/Cas9 ADAM8 (hA8) KO cell clones reveals an ADAM8-dependent regulation of miR-181a-5p. Confirmation of the CRISPR/Cas9 ADAM8 stable KO in U87 cells by qPCR **(A)**, Western Blot **(B)**, and ELISA **(C)**. **(D)** A RT-qPCR-based miRNA PCR Array (Human Finder, Qiagen) of U87 CRISPR/Cas9 ADAM8 KO cells enabling the analysis of a total of 84 miRNAs. The legend for the fold-changes in the heat map is given above representing the fold-change values (2^-ΔΔCT^) relative to U87_CTRL cells in both *ADAM8* KO clones. Note the variance in fold changes between the two KO clones **(E)** Confirmation of miR-181a-5p upregulation in U87_KO1 and U87_KO2 cells (p-value: 0.01 and 0.03). The expression of miR-181a-5p **(F)** and *ADAM8* **(G)** in GBM cell lines (G28, G112, U251), primary patient-derived cell lines (GBM29, 98, and 42), and GBM stem-like cell lines (GSCs) (GSC 2016/240, GSC 2017/74, GSC 2017/151) are shown as relative values to the expression in U87 cells. MiR-181a-5p is mostly expressed in GSCs. The patient-derived cell line GBM42 shows the highest ADAM8 expression. Pearson correlation analysis of miR-181a-5p and *ADAM8* in cell lines **(H)**, primary cell lines **(I)**, and primary GSCs **(J)** reveal a negative correlation only observed in GSCs (p-value: 0.01, Pearson r: -0.88). Results are given as mean +/- SD of two to three independent experiments. Unpaired two-tailed students *t-test* or two way ANOVA for multiple comparison **(F)** were applied to determine significance: *p < 0.05, **p < 0.01, ***p < 0.001. n.d., not detectable.

To determine potential ADAM8 correlated miRNAs, we generated stable ADAM8 knockout (KO) U87 cell clones using two guide RNAs (U87 gRNA cl. 1, U87 gRNA cl. 2) for the CRISPR/Cas9 homologous recombination method. U87 cells expressing high endogenous levels of ADAM8 were subjected to CRISPR/Cas9 induced genomic editing. After cell selection with puromycin, independent cell clones were grown and compared to U87 cells (in the following termed U87_CTRL) for morphological features and ADAM8 expression levels. From around 30 individual cell clones, two U87 gRNA clones were selected for further analyses (**Supplementary Figure 1**). Confirmation of successful ADAM8 knockout in these two U87 gRNA cell clones was provided by qPCR, Western Blot, and ELISA (**Figures 1A–C**). U87 gRNA cl. 1 and U87 gRNA cl. 2 (termed U87_KO1 and U87_KO2) showed a strong downregulation of *ADAM8* mRNA compared to U87_CTRL, p < 0.001 (**Figure 1A**). Western Blots confirmed successful ADAM8 knockout on the protein level (**Figure 1B**). In addition, ELISA measurements from cell supernatants revealed soluble ADAM8 levels below the detection limit in U87_KO clones compared to U87_CTRL (p < 0.05, **Figure 1C**). For two representative KO clones as well as a U87 control clone, a microRNA PCR Array (Human Finder) was screened. Differences in miRNA expression (given a ratio KO/CTRL) for both U87_KO clones are presented in a heatmap (**Figure 1D**) with green color representing upregulation of miRNA in U87_KO cells.

Several miRNAs were consistently upregulated in both KO clones and miR-181a-5p was selected for further investigations due to its reported regulation of osteopontin/SPP1 which also applies to ADAM8. Moreover, of all four miRNAs upregulated in U87 *ADAM8* KO cells, miR-181a-5p was the only one regulated after treatment of U87 wild-type cells with an ERK1/2 inhibitor indicating its influence in ADAM8-mediated signaling (**Supplementary Figure 2**). To further validate our miRNA screening, qPCR experiments were performed to detect miR-181a-5p expression in U87_KO and U87_CTRL cells. We confirmed upregulation of miR-181a-5p in U87_KO1 and U87_KO2 compared to U87_CTRL cells, p < 0.05 and p < 0.01, respectively (**Figure 1E**).

Next, we analyzed the expression profiles of ADAM8 and miR-181a-5p in several GBM cell lines, including U87, U251, G112, G28, three primary patient-derived cell lines GBM42, GBM29, GBM98, and three patient-derived Glioblastoma stem-like cell lines (GSCs), 2016/240, 2017/151 and 2017/74 (**Figures 1F, G**). GSCs showed low ADAM8 mRNA and high miR-181a-5p expression levels. Primary GBM cell lines showed great variability in ADAM8 and miR-181a-5p expression with GBM42 with the highest ADAM8 levels. Interestingly, knocking ADAM8 down with siRNA showed elevated levels of miR-181a-5p in GBM42 (**Supplementary Figure 3**). Pearson correlation analyses revealed exclusively in the case of GSCs a clear negative correlation of ADAM8 and miR-181a-5p expression (**Figures 1H–J**). U87_CTRL cells as well as primary GBM42 cells showed the highest endogenous ADAM8 levels in qPCR experiments compared to all other cell lines and were selected for further experiments.

### ADAM8 Regulates miR-181a-5p Expression *via* STAT3 and MAPK Signaling

**Figure 2:**
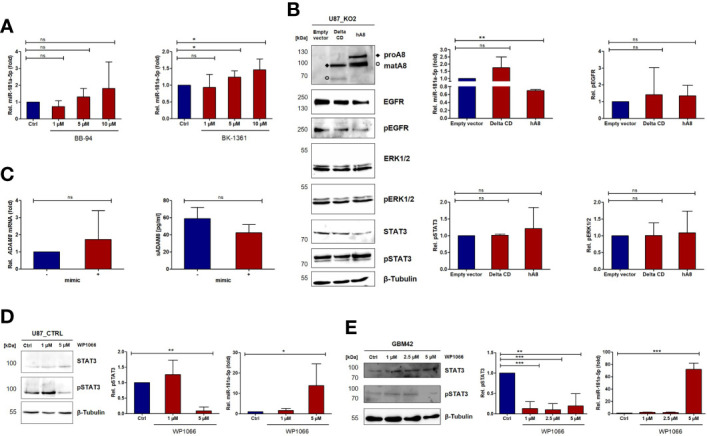
ADAM8 regulates the expression of miR-181a-5p *via* JAK2/STAT3 signaling. **(A)** U87_CTRL cells were analyzed for miR-181a-5p expression by RT-qPCR after treatment with the broad-spectrum MMP inhibitor BB-94 (left) and the ADAM8 inhibitor BK-1361 (right). **(B)** One representative western blot of three independent experiments shows the rescue of either ADAM8 lacking the cytoplasmatic domain (Delta CD) or the full-length ADAM8 (hA8). The quantifications of pEGFR, pSTAT3, and pERK1/2 are depicted on the right side and were normalized to β-Tubulin and total-EGFR/β-Tubulin, total STAT3/β-Tubulin, or total ERK1/2/β-Tubulin. Also, RT-qPCR results show no differences in miR-181a-5p expression after the transfection of ADAM8 Delta CD but a downregulation with the full-length ADAM8 rescue (p-value: 0.002). **(C)** The expression of *ADAM8* mRNA (RT-qPCR, left) and secreted ADAM8 (ELISA, right, n=2) is not affected after miR-181a-5p mimic transfection. U87_CTRL cells **(D)** and patient-derived GBM42 cells **(E)** were treated with JAK2/STAT3 inhibitor WP1066 as indicated and analyzed *via* western blot and RT-qPCR. In **(D)**, qPCR results are shown as mean values +/- SD of four independent experiments and in **(E)**, results of miR-181a-5p are described as mean values of three technical replicates. Inhibition of JAK2/STAT3 increases miR-181a-5p expression (U87 p-value: 0.027; GBM42 p-value: 0.004). Results are shown as mean values +/- SD from three independent experiments if not otherwise stated. Unpaired two-tailed students *t-test* was applied to determine significance: ns, not significant, *p < 0.05, **p < 0.01, ***p < 0.001.

To analyze the apparent ADAM8/miR-181a-5p dependence on the mechanistic level, we tested the contribution of either the metalloprotease activity or the functions of the non-proteolytic domains (DC/CD) of ADAM8 on miR-181a-5p expression. To address this, U87 cells were treated with either a broad-range metalloprotease inhibitor BB-94 (Batimastat) or with BK-1361, a selective ADAM8 inhibitor. While BB-94 did not affect miR-181a-5p expression, treatment with 10 µM and even 5 µM BK-1361 led to an increase in miR-181a-5p expression, p < 0.05 (**Figure 2A**) suggesting a contribution of the DC/CD domain on miR-181a-5p regulation by ADAM8. Moreover, we transiently re-expressed ADAM8 in U87_KO1 and analyzed the effect on miR-181a-5p expression. U87 gRNA_KO2 was transfected with either wild-type ADAM8 (hA8) or with an ADAM8 variant lacking the cytoplasmatic domain (Delta CD). Western Blots confirmed re-expression of ADAM8 variants (**Figure 2B**).

Re-expression of wild-type ADAM8 caused a downregulation of miR-181a-5p, p < 0.01 (**Figure 2B**). In contrast, cells expressing the ADAM8 delta CD variant showed no downregulation of miR-181a-5p (**Figure 2B**). These results indicate that the cytoplasmatic domain of ADAM8 triggers signaling cascades that lead to the downregulation of miR-181a-5p concomitant with a trend of increased pSTAT3 in cells transfected with wild-type ADAM8 (**Figure 2B**). Interestingly, this regulation only works in one direction, as changes in miR-181a-5p expression, i.e. by mimic transfection, do not affect expression levels of ADAM8 in U87 cells (**Figure 2C**). We explored the role of two downstream signaling pathways of ADAM8 CD, STAT3 signaling and MAPK signaling. For this purpose, U87 cells and primary GBM cells GBM42 were treated with either U0126 (MEK1/2 inhibitor) or WP1066 (STAT3 inhibitor). MEK1/2 inhibition caused an increase in miR-181a-5p expression in U87_CTRL cells (p < 0.05), and a tendency to increase in primary GBM42 cells (p-value: 0.052) (**Supplementary Figure 4**). More prominently, STAT3 inhibition by WP1066 was confirmed for both cell lines *via* western blot and resulted in increased expression levels of miR-181a-5p in both cell lines with p < 0.05 (**Figures 2D, E**).

### MiR-181a-5p Regulates Cell Proliferation and MMP9 Expression

**Figure 3:**
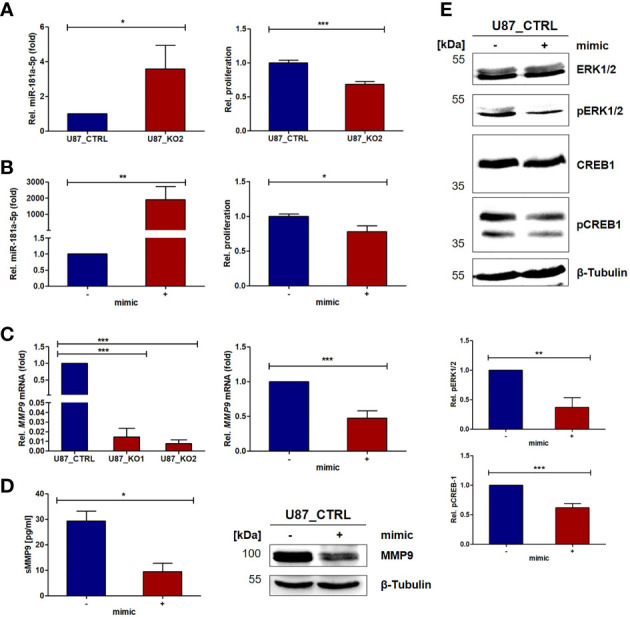
ADAM8 affects MMP9 expression and proliferation *via* miR-181a-5p targeting ERK2 and CREB-1. **(A)** The upregulation of miR-181a-5p in U87_KO cells (RT-qPCR, n=3, left) causes inhibition of proliferation (CellTiter Glo, n=2, measurement after 48 h, right). **(B)** Overexpressing miR-181a-5p in U87_CTRL cells *via* transient transfection (RT-qPCR, n=3, left) causes inhibition of proliferation after 48 h (CellTiter Glo, n=3 technical replicates, right). **(C)** MMP9 is downregulated in U87_KO cells on mRNA (RT-qPCR, n=3, left). After miR-181a-5p mimic transfection, MMP9 is downregulated in U87 cells on the mRNA (RT-qPCR, n=3, p-value: 0.0009) **(C)** and on protein level (ELISA, n=2, p-value: 0.0004) analyzed by western blot **(D)**. **(E)** Analyses of kinase activation after miR-181a-5p mimic transfection for ERK1/2 (p-value: 0.003) and CREB-1 (p-value: 0.0007). Results are shown as mean values +/- SD of three independent experiments unless otherwise stated. Results are given in mean +/- SD. Unpaired one-tailed students *t* test was applied to determine significance: *p < 0.05, **p < 0.01, ***p < 0.001.

We further analyzed whether miR-181a-5p can affect the cell proliferation of GBM cells. Exemplified for U87_KO2, a decrease in cell proliferation was observed (p < 0.001, **Figure 3A**). This effect can be recapitulated when mimic miR-181a-5p was transfected into U87 cells (p < 0.01, **Figure 3B**). As an oncoprotein able to promote GBM cell proliferation, we analyzed *MMP9* expression in U87_KO2 and mimic transfected U87 cells (**Figures 3C, D**) (27). *MMP9* mRNA levels in U87_KO2 and mimic transfected cells are strongly downregulated as revealed by qPCR (p < 0.001, **Figure 3C**). After mimic miR-181a-5p transfection of U87 cells, ELISA experiments revealed less soluble MMP9 levels in cellular supernatants (p < 0.05, **Figure 3D**). Comparable results were obtained for osteopontin (**Supplementary Figure 7**). Next, we explored whether miR-181a-5p dependent MMP9 downregulation is a result of direct miR-181-a/*MMP9* mRNA interaction. Three target prediction tools, miRDB, TargetScan, and TargetMiner, predicted no miR-181a-5p binding site. Also, bioinformatic analysis of the MMP9 3’ UTR did not reveal a sufficiently long binding site for miR-181a-5p. Thus, we conclude that MMP9 is most likely indirectly regulated by miR-181a-5p. Indeed, literature research and the utilization of the target prediction tools miRDB and TargetScan revealed that miR-181a-5p directly targets three kinases of the MAPK pathway, CREB-1, MEK1, and ERK2 (**Supplementary Table 1**, 38, 39). To demonstrate that, transfection of U87_CTRL cells with a miR-181a-5p mimic was performed and revealed downregulation of pERK1/2 and p-CREB-1 in three independent Western Blot experiments, with p < 0.01 and p < 0.001, respectively (**Figure 3E**). Notably unphosphorylated levels of ERK1/2 and CREB-1 were not influenced by mimic transfection (**Figure 3E**). Thus, our results further support ERK2 and CREB-1 as downstream targets of miR-181a-5p.

### EVs Derived From U87_KO Cells Are Associated With Higher miR-181a-5p Levels

**Figure 4:**
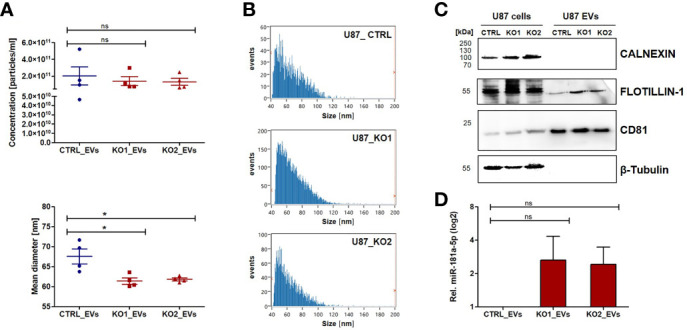
ADAM8 affects miRNA181a-5p levels associated with EVs in U87 cells. **(A)** U87_CTRL and U87_KO derived EVs were characterized regarding their concentration (upper graph) and size (lower graph). Measurements were taken with the NanoFCM device and results are shown as mean values +/- SEM of four independent experiments. **(B)** Representative size distributions of U87_CTRL and U87_KO derived EVs (NanoFCM) are shown in bar graphs. **(C)** *Via* western blot, the presence of EVs was confirmed. CALNEXIN was used as negative control and FLOTILLIN-1 as well as CD81 as EV markers. **(D)** MiR-181a-5p is detectable in U87 derived EVs. RT-qPCR results show a non-significant tendency of miR-181a-5p upregulation in U87_ KO derived EVs. Mean values +/- SD of three independent experiments if not otherwise stated. Unpaired two-tailed students *t-test* was applied to determine significance: ns, not significant, *p < 0.05.

Having demonstrated the intracellular effects of ADAM8 on miR-181a-5p and *MMP9* as a target gene, we further investigated whether EVs derived from cellular supernatants of U87_CTRL (CTRL_EVs), U87_KO1 (KO1_EVs), and U87_KO2 (KO2_EVs) are associated with miR-181a-5p expression. By Nanoflow Cytometry Measurement (NanoFCM), the size and concentration of EVs prepared from cellular supernatants were analyzed (**Figures 4A, B**). Western Blot experiments further confirmed the presence of EVs using FLOTILLIN-1 and CD81 as EV markers, CALNEXIN as a negative control, and ß-Tubulin as a predominant lysate marker (**Figure 4C**). MiR-181a-5p was detected in all three EV populations (CTRL_EVs, KO1_EVs, KO2_EVs) and consistent with our observation in U87_CTRL and U87_KO cells, KO1_EVs and KO2_EVs displayed higher miR-181a-5p levels than CTRL_EVs (**Figure 4D**). To ensure that more miR-181a-5p is packed in EVs with higher cellular expression, we separated EVs from ctrl and mimic transfected cells with a 28-fold enrichment of miR-181a-5p in EVs derived from mimic transfected cells (**Supplementary Figure 8**). Furthermore, we confirmed the uptake of KO2_EVs by U87_CTRL cells *via* immunofluorescent microscopy by incubating CFSE-stained KO2_EVs as well as CTRL_EVs with Hoechst-stained U87_CTRL cells (**Supplementary Figure 9A**). Western blot analysis showed downregulation of MMP9 in U87_CTRL cells incubated with both CTRL_EVs and KO2_EVs in comparison to the HBSS control but did not confirm a significant difference of MMP9 expression comparing cells incubated with KO2_EVs or CTRL-EVs (**Supplementary Figure 9B**). We treated U87_KO2 cells with either miR-181a-5p-mimics or a miR181a-5p inhibitor and incubated the corresponding EVs with U87_CTRL cells. An ELISA experiment revealed that incubation of inhibitor-treated EVs led to increased soluble MMP9 levels whilst incubation of miR-181a-5p-mimic treated EVs caused a decrease in soluble MMP9 (**Supplementary Figure 9C**).

### Characterization of ADAM8, MMP9, and miR-181a-5p Expression in GBM Tumor Tissue Samples

**Figure 5:**
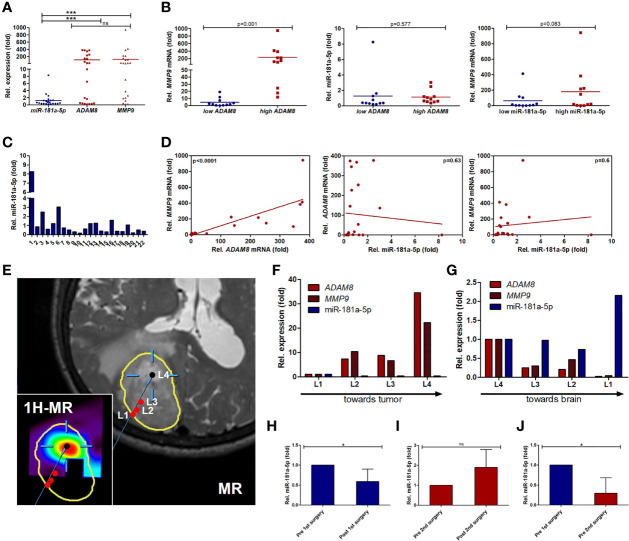
Expression levels of *ADAM8* and *MMP9*, and miR-181a-5p in GBM tissue samples. **(A)** RT-qPCR results of GBM tissue samples (n=22, fold change normalized to 1) indicate a higher expression of *ADAM8* (p-value: 0.0009) and *MMP9* (p-value: 0.0002) than miR-181a-5p. **(B)** Dividing the RT-qPCR results and patient cohort into two groups (low/high *ADAM8* or low/high miR-181a-5p expression) reveals a correlation of *ADAM8* with *MMP9* (left graph, p-value: 0.001) but no correlation of miR-181a-5p with *ADAM8* (middle graph, p-value: 0.577) or *MMP9* (right graph, p-value: 0.083) expression. **(C)** RT-qPCR results for miR-181a-5p expression of each GBM tissue sample. **(D)** *ADAM8* and *MMP9* are correlated in GBM tissue samples (p < 0.0001, n=22), whereas the inverse correlations of *ADAM8* and miR-181a-5p and of miR-181a-5p and *MMP9* are non-significant (p-values: 0.63 and 0.6, respectively). **(E)** T2-weighted magnetic resonance (MR) image showing a left parietal GBM (segmented in yellow, patient 25) as well as the co-registered choline/N-acetylaspartate (NAA) maps derived from 1H-MR spectroscopy, integrated into the neuronavigation system for navigated extraction of tissue samples (L1: tumor border, L2/L3: tumor, L4: tumor, Cho/NAA hotspot) magnetic resonance (heatmap for choline metabolite). Corresponding molecular analyses are shown in **(F, G)** (patient 25). RT-qPCR results of *ADAM8* (red), *MMP9* (tiled red), and miR-181a-5p (blue) in different tissue locations normalized to either L1 **(F)** or L4 **(G)** describing the direction of surgery. **(H)** In a pilot study, three GBM patients (Patient 9, 23, 24) were analyzed for their serum-EV miR-181a-5p expression *via* RT-qPCR. The serum was collected before and after the first and second surgery. Interestingly, after first surgical resection miR-181a-5p is less expressed in serum-EVs (p-value: 0.042). **(I)** After second surgery, miR-181a-5p shows a slight increase in serum-EVs (p-value: 0.08). **(J)**, miR-181a-5p is less detectable in serum-EVs prior to the second surgery compared to pre-first surgery (p-value: 0.02; left graph). Results are shown in mean values +/- SD. Unpaired one-tailed students *t* test and Wilcoxon signed-rank test were applied to determine significance: ns, not significant, *p < 0.05, **p < 0.01, ***p < 0.001.

RT-qPCR experiments were conducted on 22 tumor tissue samples from patients admitted to our clinical department to analyze the expression profiles of *ADAM8*, *MMP9*, and miR-181a-5p in GBM tissue. Further information on the patient cohort and histopathological data are listed in **Table 1**. For normalization of data (set to 1 in **Figures 5A–D**), we utilized tissue samples localized most remote from the tumor core. The majority of the examined tumor tissue samples showed downregulation of miR-181a-5p (**Figure 5C**). In contrast, mean *ADAM8* and *MMP9* expression levels were upregulated in the investigated tumor samples (**Figure 5A**). High *ADAM8* correlated with elevated *MMP9* expression levels, p < 0.0001 (**Figure 5D**). In the patient cohort, neither *ADAM8* mRNA levels nor *MMP9* mRNA levels correlated with miR-181a-5p expression, p = 0.6 and p = 0.63 respectively (**Figure 5D**). We then divided the patient cohort into subgroups, high *ADAM8* expression, and low *ADAM8* expression group, as well as high miR-181a-5p expression and low miR-181a-5p expression group (**Supplementary Figure 9**). *MMP9* expression was elevated in the high *ADAM8* group, p = 0.01 (**Figure 5B**). MiR-181a-5p expression was similar in the high *ADAM8* and low *ADAM8* groups (**Figure 5B**). *MMP9* expression was also similar in both miR-181a-5p subgroups (**Figure 5B**). To further investigate if this trend is due to the strong heterogeneity of the GBM tissue, we explored the connection between ADAM8, MMP9, and miR-181a-5p in a pilot experiment using MR-spectroscopy guided surgery at different locations in a GBM tumor tissue sample of one selected patient. In the non-tumorous access tissue (L1), miR-181a-5p showed the highest expression whereas *ADAM8* and *MMP9* expression is at the lowest level (**Figures 5E–G**). Analysis of tumor edge (L2 and L3) and core tumor (L4) with strongly proliferating and vascularized zones revealed reversed expression patterns for *MMP9*, *ADAM8*, and miR-181a-5p (**Figures 5E–G**). Tumor locations in L3 and L4 were also confirmed by 1H-MR spectroscopy (**Supplementary Table 2**).

### MiR-181a-5p Expression in Serum-Derived EVs From GBM Patients

In a further pilot study, serum specimens from three GBM patients were obtained before and after surgical resection. All three patients suffered from tumor recurrence and underwent surgical resection for a second time. In all cases, the highest miR-181a-5p expression levels were observed in serum samples prior to the first surgical resection (**Figure 5H**). After the first surgery, a reduction in miR-181a-5p levels was observed in post-surgery serum-derived EVs, p < 0.05 (5H). In contrast, after the second surgery, miR-181a-5p expression was slightly upregulated (**Figure 5I**). A comparison of primary manifested GBM and recurrent GBM revealed a decrease in miR-181a-5p expression in EVs, p < 0.05 (5K). These results suggest that miR-181a-5p could serve as a tumor marker, but needs to be sufficiently powered in further studies.

## Discussion

**Figure 6:**
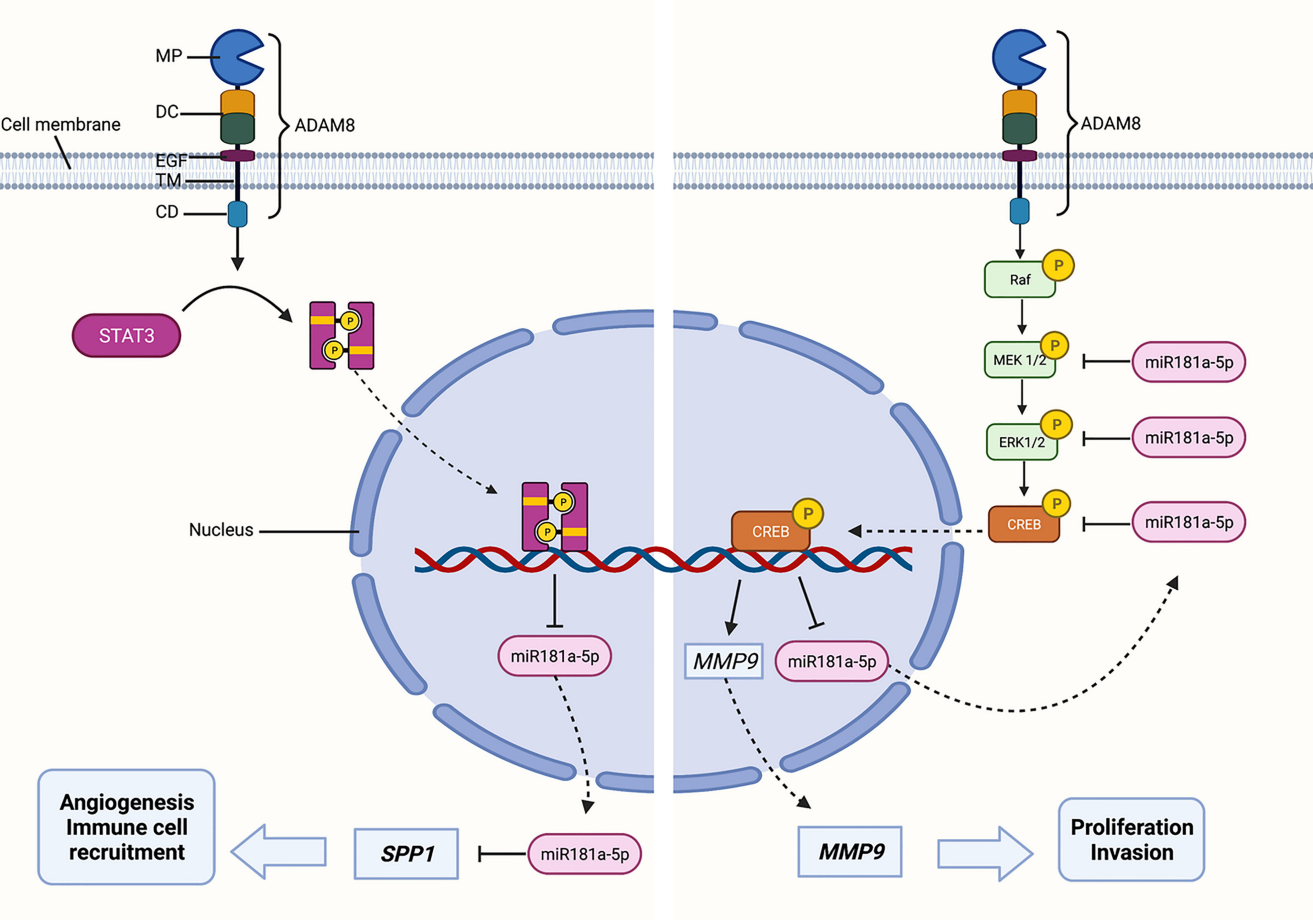
Sketch of ADAM8-dependent effects caused by regulation of miR-181a-5p in GBM cells. ADAM8 with homologous domains including the metalloprotease domain (MP), the disintegrin/cysteine-rich domain (DC), the EGF-like domain (EGF), the transmembrane (TM), and the cytoplasmic domain (CD). ADAM8 activates intracellular signaling cascades by STAT3 and MAPK in the presence of the cytoplasmic domain. ADAM8/STAT3/miR-181a/*SPP1* axis: ADAM8 dependent STAT3 activation downregulates miR-181a-5p, as miR-181a-5p targets *SPP1*, disinhibition of *SPP1* leads to several tumor progressing effects such as induction of angiogenesis and enhanced immune cell recruitment. ADAM8/MAPK/MMP9 axis: ADAM8 activates the MAPK pathway, the transcription factor CREB-1 induces MMP9 transcription and inhibits miR-181a-5p transcription. MMP9 promotes tumor cell proliferation and invasion. By targeting CREB-1, ERK2, and MEK1, miR-181a-5p downregulates MMP9 expression most likely by an indirect mechanism. Created with BioRender.com.

ADAM8 as a multidomain enzyme exhibits numerous tumor-supporting characteristics by promoting invasion, angiogenesis, and chemoresistance in GBM (10, 12). Due to these multiple functions, ADAM8 affects several intracellular pathways involving several important kinases and transcription factors such as JAK2/STAT, AKT/PI3K, ERK1/2, and CREB-1 (8–12). Mechanistically, the ADAM8 metalloprotease domain cleaves extracellular membrane components while the cytoplasmatic domain activates crucial signaling cascades in carcinogenesis (4). Thereby, ADAM8 induces the expression of several oncoproteins including MMP9 and *SPP1*/osteopontin (8, 10). Previously, we demonstrated that ADAM8-dependent MMP9 expression is mediated *via* the MAPK pathway and resulted in a strong correlation of ADAM8 and MMP9 in breast cancer-derived brain metastasis (8). By characterizing the expression profile of ADAM8 and MMP9 in GBM tissue samples, we confirmed these observations for GBM. To dissect the effects of ADAM8 on oncoproteins mechanistically, we hypothesized that ADAM8 could alter the expression levels of distinct miRNAs such as miR-720, as previously shown for breast cancer cells (13). Generation of stable ADAM8 KO clones with subsequent miRNA screening revealed that the tumor suppressor miRNA miR-181a-5p shows a significantly higher expression in GBM cells deficient in ADAM8. Since high ADAM8 levels are correlated with GBM progression, a downregulation of miRNA181a-5p would be expected. Indeed, a recent study linked the poor prognosis of GBM patients with low miRNA181a-5p expression levels (40). Together, these findings qualified miRNA181a-5p as a candidate for a detailed molecular analysis, as presented here. Transient re-expression of ADAM8 in U87_KO cells resulted in downregulation of miR-181a-5p, suggesting that ADAM8 actively suppresses the expression of miR-181a-5p. Downregulation of miR-181a-5p by ADAM8 is dependent on the presence of the cytoplasmatic domain. In GBM, miR-181a-5p acts as a tumor suppressor miRNA by reducing invasiveness and enhancing radio- and chemosensitivity (23, 41). We confirmed that overexpression of miR-181a-5p led to reduced proliferation rates in U87 cells. Moreover, a similar effect on cell proliferation was observed in ADAM8 deficient GBM cells. It was shown that miR-181a-5p suppresses cell colony formation and tumor growth, and regulates apoptosis by targeting BCL-2 (23, 41). It is interesting to note that GSCs express relatively high levels of miRNA181a-5p compared to differentiated GBM cells, which could be instrumental in regulating proliferation and cell survival of this particular cell type. We have evidence that ADAM8 and, negatively correlated, miRNA181a-5p levels change in GSCs under conditions favoring differentiation of GSCs (Schäfer, unpublished data). However, the mechanisms that lead to miR-181a-5p downregulation in GBM remained elusive until now. As ADAM8 is a membrane-anchored protein, we concluded that ADAM8 downregulates the expression of miR-181a-5p by downstream signaling and activation of transcription factors. Indeed, our results revealed that miR-181a-5p can be downregulated by the activation of STAT3 and MAPK pathways. Conversely, miRNA181a-5p can regulate either total STAT3 levels in U87 cells and, notably, affect levels of p-STAT3 in the primary GBM cell line GBM42, indicating an unknown mechanism of kinase regulation by miRNA, similar to an observation made for phospho-AKT and p-ERK in a previous study in glioma (42) Previously, the importance of STAT3 signaling in GBM has been demonstrated in numerous studies whilst our group showed that ADAM8 dependent activation of STAT3 signaling led to increased angiogenesis by upregulation of osteopontin (10, 43, 44, reviewed in 45). In agreement with these findings, the 3’UTR of *SPP1*/osteopontin contains a binding site for miR-181a-5p and can be downregulated upon miR-181a-5p overexpression (19). All these results support the existence of a possible ADAM8/STAT3/miR-181a/osteopontin axis in GBM (**Figure 6** left). In addition, increased activation of the MAPK pathway is observed in numerous malignant tumors and leads to uncontrolled cell growth and mitosis (46). One of the best-known activators of the MAPK signaling pathway is the EGFR. Frequently, primary GBM tumors display a constitutively active variant, EGFRvIII (47). Apart from EGFR dependent MAPK activation, ADAM8 can activate the MAPK pathway EGFR independently (9). Interestingly, two kinases of the MAPK pathway, ERK2, and MEK1 as well as the downstream transcription factor CREB-1 are known to contain binding sites for miR-181a-5p (25, 26). In our experiments, phosphorylated and thus activated pCREB and pERK1/2 were downregulated in U87 cells transfected with miR-181a-5p mimics. We did not observe any effects on unphosphorylated CREB-1 and ERK1/2 as well as on MEK1/2 expression. Since miRNAs are post-transcriptional regulators of protein expression, we do not fully understand these results, but a TargetScan search revealed that CRBL2, a protein regulating phosphorylation of CREB1 is directly regulated by miR181a-5p, adding one more level of complexity to the network we have described here. A study by Fu et al. showed that CREB-1 suppresses miR-181a-5p transcription by directly binding to its promoter region (48). Thus, the interaction of miR-181a-5p and the MAPK pathway may constitute a regulatory loop that requires further investigation. Furthermore, we can postulate that our results describing the regulation of miR-181a-5p by ADAM8 are not restricted to the role of ADAM8 in GBM, as all other tumor cell lines that we investigated so far such as the triple-negative breast cancer cell line MDA-MB-231 and the PDAC cell line Panc89 show elevated levels of miR-181a-5p upon ADAM8 deficiency (unpublished observations). In accordance, MDA-MB-231 were among those cell lines that showed a strong correlation between ADAM8 and MMP9 expression in our previous study on breast cancer-derived brain metastases (8).

MMP9 plays a central role in tumor progression, especially for cell proliferation and invasion (27). Moreover, MMP9 expression is a prognostic factor in GBM and negatively correlated with patient survival (30). Thus, exploring the miR-181a-5p dependent *MMP9* downregulation was particularly interesting. U87 cells overexpressing miR-181a-5p exhibited decreased MMP9 levels. This was observed in U87 ADAM8 knockout cells as well as U87_CTRL cells incubated with miR-181a-5p mimics. To further establish whether *MMP9* mRNA contains a binding site for miR-181a-5p, we utilized target prediction tools and analyzed the mRNA sequence of MMP9. However, this analysis revealed that the *MMP9* mRNA does not contain an authentic binding site for miR-181a-5p. Consequently, we concluded that miR-181a-5p can indirectly downregulate MMP9 expression by silencing the MAPK cascade (**Figure 6** right). This conclusion is supported by data showing that ERK1/2 inhibition led to decreased MMP9 levels in U87 and GBM42 cells (**Supplementary Figure 5**).

All these results demonstrate intracellular regulatory mechanisms of ADAM8/miR-181a-5p signaling so far. Cell-cell communication in the tumor microenvironment is essential for shaping either an immunosuppressive or a tumor-supportive microenvironment (34). As one mode of cell-cell communication, tumor cells release EVs. These heterogeneous nanoparticles contain a great variety of different molecules including miRNAs (35). Clinically, EVs received increasing attention, as their function as novel diagnostic and prognostic biomarkers is discussed (49). In our study, we analyzed the miR-181a-5p expression in U87 cells and serum-derived EVs. We recapitulated the higher abundance of miR-181a-5p in EVs from ADAM8 KO cells. Concerning patient sera, miR-181-5p expression in EVs dropped after the first surgical tumor resection. Moreover, miR-181a-5p expression was downregulated in serum-derived EVs from recurrent GBM. These results suggest that miR-181a-5p is further downregulated along with tumor progression. However, additional analyses must be carried out in a larger patient cohort to support this conclusion. The uptake of EVs can alter the behavior of recipient cells (50). Therefore, EVs might also be utilized as therapeutic vehicles (51). In our experiments, miR-181a-5p enriched vesicles were taken up by naive U87 cells demonstrating a role for ADAM8 in the tumor microenvironment. It remains to be determined if GBM resident immune cells such as macrophages that constitutively express ADAM8 could release EVs that might fail to suppress MMP9 expression in target cells, in conjunction with the possible tumor-promoting role of ADAM8 in macrophages (33).

Due to limited therapeutic options as well as the absence of early diagnostic biomarkers, GBM remains challenging as an incurable disease with a grim prognosis. Therefore, the identification of potential biomarkers as well as new therapeutic targets is of high importance. In summary, we identified that ADAM8 downregulates miR-181a-5p by activation of STAT3 and MAPK signaling. Considering that miR-181a-5p is a tumor suppressor miRNA in GBM, ADAM8 dependent silencing of miR-181a-5p could further contribute to tumor progression. We showed that overexpression of miR-181a-5p decreased cell proliferation and suppressed MMP9 expression by downregulation of the MAPK pathway Moreover, the presence of miR-181a-5p in clinical samples and EVs isolated from cellular supernatants as well as patient sera justifies further studies to reveal a potential role of miR-181a-5p in GBM diagnosis and progression.

## Data Availability Statement

The raw data supporting the conclusions of this article will be made available by the authors, without undue reservation.

## Ethics Statement

The studies involving human participants were reviewed and approved by Local Ethics Committee (Philipps University Marburg, medical faculty, file number 185/11). The patients/participants provided their written informed consent to participate in this study.

## Author Contributions

JWB, ES, BC, CN, and MB conceived this study. AS, LE, LM, US, GLD, ABB, OL, and CP performed experiments and evaluated the data. ACB, MP, CN, and BC provided resources and clinical data. AS, LE, and JWB wrote the manuscript draft. MB, CN, and MP reviewed and edited the manuscript. All authors contributed to the article and approved the submitted version.

## Funding

Work was supported in the framework of ERANET PerMed joint call 2018, project PerProGlio by the Federal Ministry for Education and Research (BMBF), grant number 01KU1915B to JWB, AS and by the Deutsche Forschungsgemeinschaft (DFG) grant BA1606/3-1 to US and JWB, and GRK 2573/1 to ES, and by a Research Grant from the University Medical Center Giessen and Marburg (UKGM). Open Access was kindly supported by the University of Marburg and the DFG. 

## Conflict of Interest

The authors declare that the research was conducted in the absence of any commercial or financial relationships that could be construed as a potential conflict of interest.

## Publisher’s Note

All claims expressed in this article are solely those of the authors and do not necessarily represent those of their affiliated organizations, or those of the publisher, the editors and the reviewers. Any product that may be evaluated in this article, or claim that may be made by its manufacturer, is not guaranteed or endorsed by the publisher.
