# Supplementary material for: The Metalloprotease-Disintegrin ADAM8 Alters the Tumor Suppressor miR-181a-5p Expression Profile in Glioblastoma Thereby Contributing to Its Aggressiveness
Source: Front Oncol. 2022 Mar 15;12:826273. doi: 10.3389/fonc.2022.826273 (PMC8964949; doi:10.3389/fonc.2022.826273)
Supplement: Supplementary file 1 [file DataSheet_1.docx]

Supplementary Material

# Supplementary Materials and Methods

## Real-Time Quantitative Polymerase Chain Reaction (qPCR) Primers

For RT-qPCR analysis, primers with the following sequences were used ordered from Apara-bioscience GmbH (Germany):

*CXCL8* fw 5’ GAA CTG AGA GTG ATT GAG AGT GGA 3’

rev 5’ CTC TTC AAA AAC TTC TCC ACA ACC 3’

*MMP14* fw 5’ GGC TAC AGC AA ATG GCT ACC 3’

rev 5’ GAT GGC CGC TGA GAG TGA C

*SPP1* fw 5’ GCC GAG GTG ATA GTG TGG TT 3’

rev 5’ TGA GGT GAT GTC CTC GTC TG 3’

For miRNA RT-qPCR, primers were obtained from Qiagen (miScript Primer Assays, Qiagen, Germany).

## Transient ADAM8 knockdown in primary GBM42 cells

To knockdown ADAM8 expression in primary GBM42 cells, cells were seeded in a 6-well-plate format at a density of 500,000 cells in 2 ml. After 24 h, transfection was performed using 0.01 µM ON-TARGET plus non-targeting Control Pool (Dharmacon, US) as control siRNA or 0.01 µM siRNA against ADAM8 (L-004502-01-005, Dharmacon, US) To knockdown the ADAM8 expression. Therefore, Lipofectamine RNAimax (Invitrogen, UK) was utilized according to the manufacturer’s protocols. Cells were harvested and analyzed by RT-qPCR and western blot after 48 h of transfection.

## Inhibitors

U0126 was purchased from cell signaling (Cat. No. 9903) and used as a selective MEK1/2 inhibitor, whereas FAK was selectively inhibited by FAK Inhibitor 14 (Cat. No. 3414, Tocris, UK). Cells were seeded in a 6-well-format (500,000 cells in 2 ml) and harvested 16 h after treatment for western blot analysis. The concentrations used are indicated in the graph.

## Enzyme-linked Immunosorbent Assay (ELISA) for soluble Osteopontin

Soluble Osteopontin (DY1433, R&D Systems, UK) from cell culture supernatants was determined by Sandwich-ELISA method with DuoSet ELISA Kit according to the manufacturer’s instructions.

## CFSE staining of EVs and incubation with U87 cells

Prior to the staining, the EV amount was determined with NanoPhotometer® NP80 (Implen GmbH, Germany). 30 µg EVs were stained with 40 µM Carboxyfluorescein succinimidyl ester (CFSE, C34570, Thermo Fisher Scientific, US) for 10 min at 4°C. As a negative control, HBSS (Gibco^TM^, Life Technologies, US) without EVs was also incubated with CFSE in the same condition and also further treated equally. EVs were pelleted via ultracentrifugation at 100,000 g for 90 min at 4°C and washed with HBSS before an additional ultracentrifugation step (100,000 g, 90 min, and 4°C). U87_CTRL cells were seeded on a coverslip in a 24-well format (100,000 in 500 µl) overnight. The coverslip was coated before with 50 µg/mL Collagen, Type I from rat tail (C7661, Sigma-Aldrich, US) for 1 hour at 37°C followed by several washing steps with PBS. Next, the CFSE stained EVs, HBSS with CFSE or only cell culture medium was added to U87 cells in a final volume of 400 µl medium per well. After 5 h of incubation, cells were washed with PBS and fixed with 4 % (w/v) paraformaldehyde in PBS for 15 min at RT. After that, cells were washed three times with PBS and permeabilized with 0.3 % (v/v) Triton-X-100 (T8787, Sigma, Germany) in PBS for 15 min followed by blocking with 5 % BSA (A7030, Sigma, Germany) in PBS. Then, cellular nuclei were stained with Hoechst 33342 dye (Sigma-Aldrich, US) diluted 1:10,000 in PBS for 20 min at RT. Images were taken using the all-in-one Fluorescence Microscope BZ-X810 (KEYENCE, Germany).

## Separation of EVs associated with miR-181a-5p mimic or inhibitor

To transiently overexpress miR-181a-5p and thereby achieve an enriched association of miR-181a-5p and EVs or to achieve a degradation and inhibition of miR-181a-5p in cells and EVs, U87 cells were seeded in 6-well-plates at a density of 400,000 cells in 2 ml and were transfected with 0.01 µM miR-181a-5p mimic (miScript, Qiagen) or 0.01 µM miRCURY LNA miR-181a-5p inhibitor (miRCURY, Qiagen) in the serum-free medium after 24 h. Transfection was performed utilizing Lipofectamine RNAimax (Invitrogen, UK) according to the manufacturer’s instructions. After 48 h, supernatants were collected and EVs were separated from two pooled wells of a 6-well-plate according to 2.7 Separation of extracellular vesicles (EVs). The whole amount of separated EVs was incubated with prior seeded and adherent cells for 24 h. Subsequently, protein lysates were generated according to 2.10 Protein Extraction and Western Blot Analysis, and supernatants were collected for MMP9 ELISA measurements according to 2.11 Enzyme-Linked Immunosorbent Assay (ELISA).

# Supplementary Figures and Tables

## Supplementary Figures


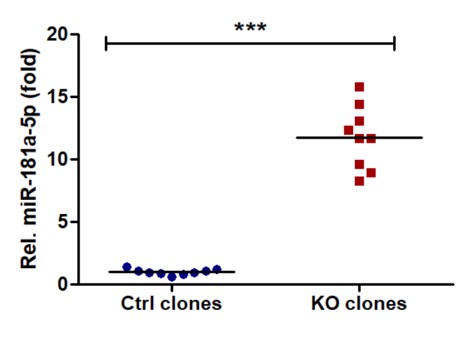


**Supplementary Figure 1.** MiR-181a-5p screening in 10 different U87 ctrl clones and 10 ADAM8 KO clones. Note that in every case miR-181a-5p is upregulated with a knockout for *ADAM8*. Paired student’s *t* test was applied for determining significance (p < 0.001).


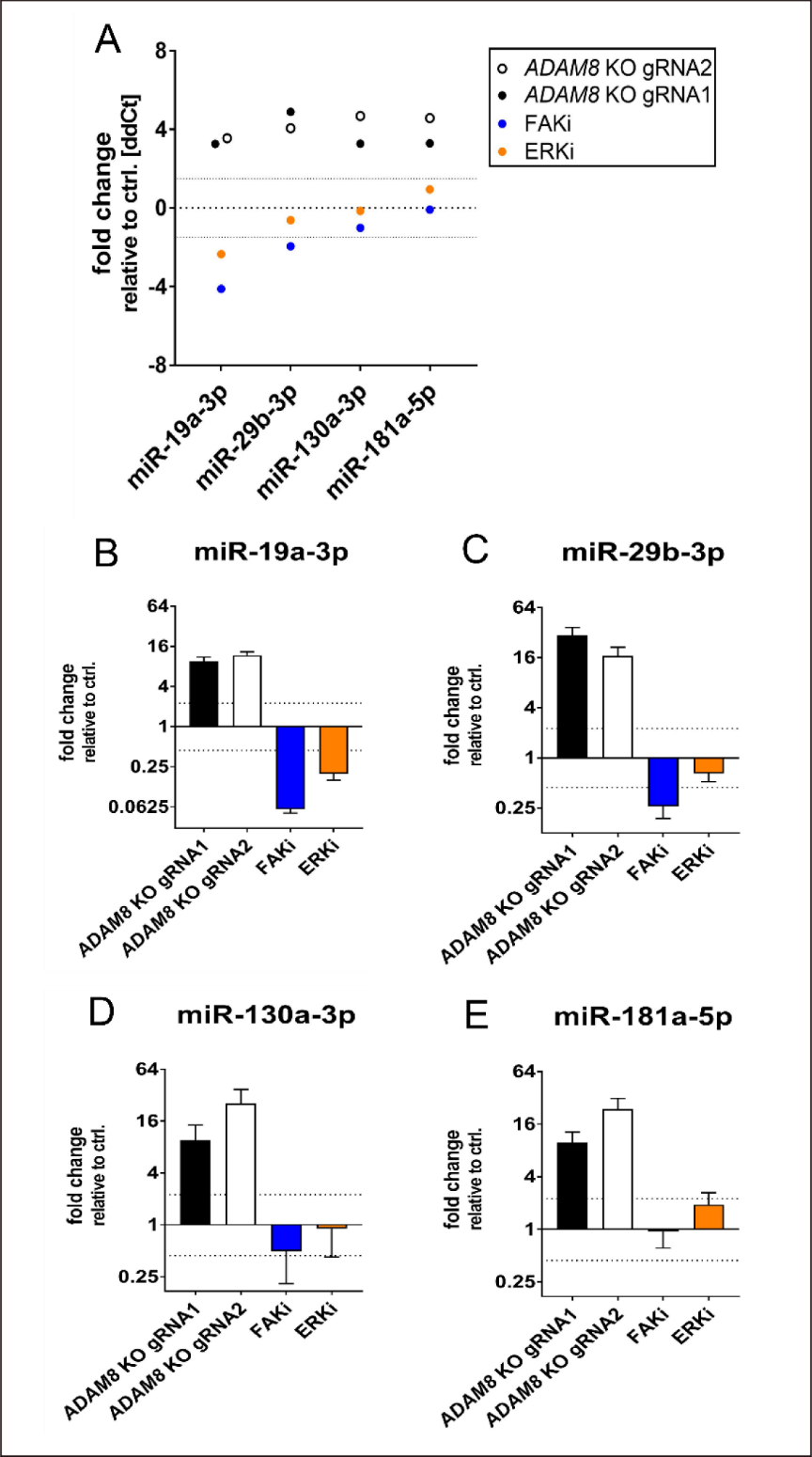


**Supplementary Figure 2.** Validation of ADAM8-dependent miRNAs identifies miR-19a, miR-29b, miR-130a, and miR181a-5p as potential tumor suppressor miRNAs in U87. Same cDNA of the microarray conditions (U87 ADAM8 expressing control cells, ADAM8 KO clones gRNA1 and gRNA2 as well as U87_CTRL cells incubated with either FAK [1 µM] or ERK1/2 [5 µM] inhibitor was subjected to RT-qPCR for validation of indicated miRNAs. (**A**) The fold change of four miRNAs is presented relative to ADAM8 expressing control cells. (**B**-**E**) Relative expression of four identified miRNAs presented as fold change (log2). A threshold indicating a significantly strong dysregulation by the dotted line was chosen marking an increase of 1.5-fold. Note that just in the case of miR-181a-5p (E) an upregulation of expression was achieved by using an ERK inhibitor.


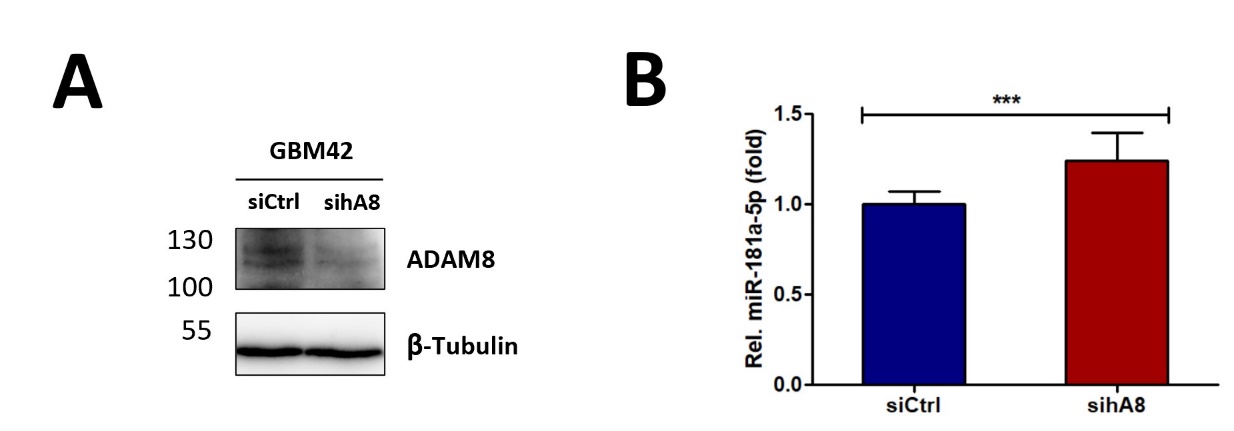


**Supplementary Figure 3.** Transient transfection of primary GBM42 cells with a siRNA against ADAM8 leads to an increase of miR-181a-5p expression. (**A**) The transient ADAM8 knockdown was confirmed via western blot analysis. (**B**) With ADAM8 knockdown in primary GBM42 cells, miR-181a-5p increases slightly.


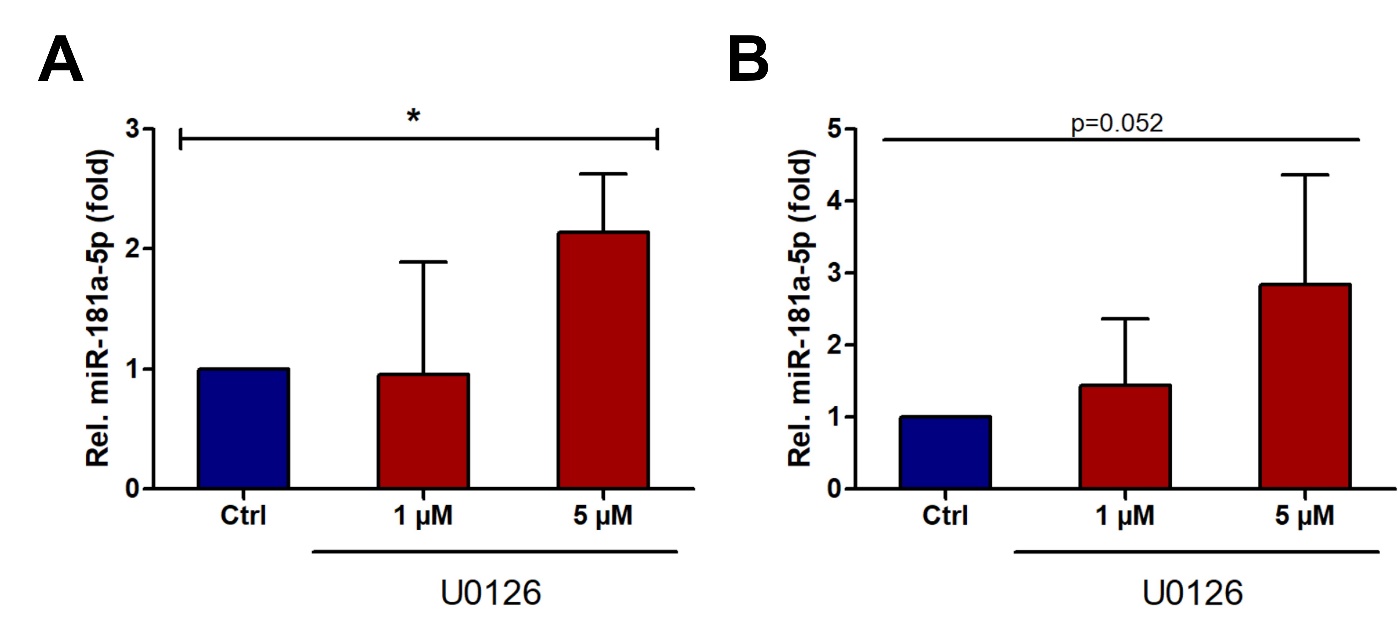


**Supplementary Figure 4.** MEK1/2 inhibition via U0126 affects miR-181a-5p expression in U87_CTRL and slightly in GBM42 cells. RT-qPCR analysis of miR-181a-5p expression in U87_CTRL (**A**) and GBM42 (**B**) cells. Results of three independent experiments. Unpaired one-tailed students t-test was applied to determine significance: * p < 0.05.


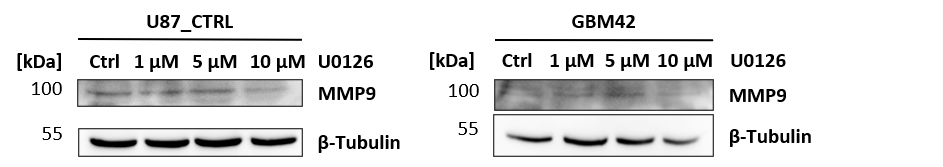


**Supplementary Figure 5.** Protein expression of MMP9 in U87 and GBM42 cells after U0126 treatment. Cells were treated with U0126 for 16 h before protein isolation. One representative western blot is shown, respectively.


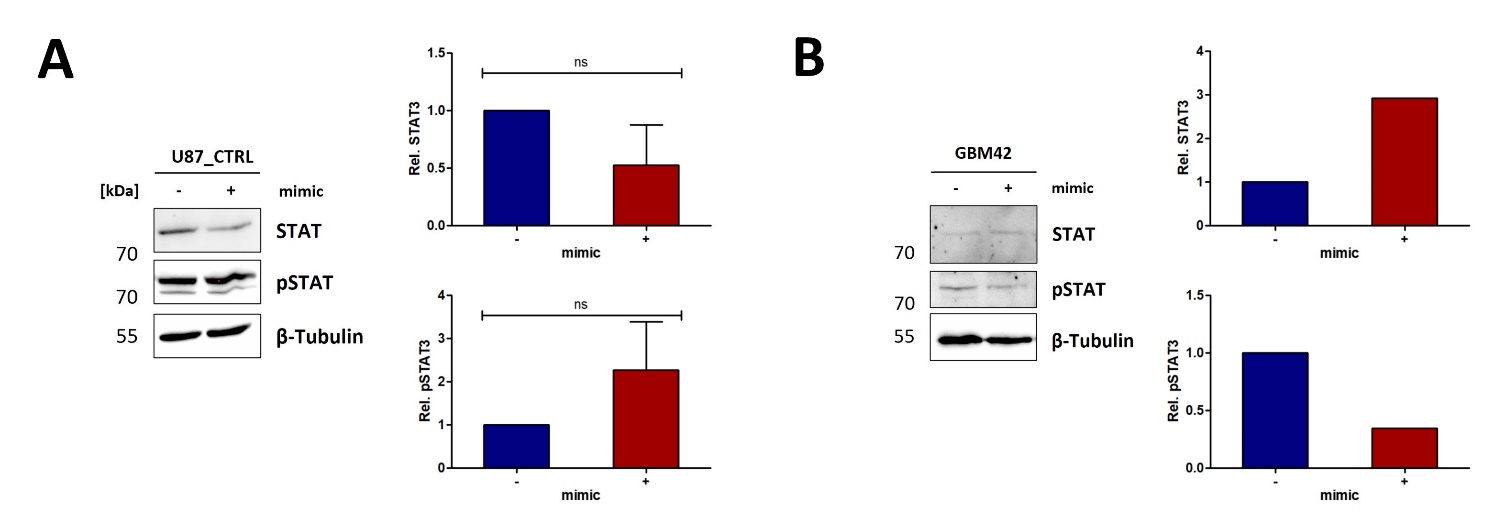


**Supplementary Figure 6.** Western Blot Analysis for STAT3 and pSTAT3 after miR-181a-5p mimic transfection in U87 cells. Graphs showing the mean quantification +/- SD of three independent experiments. Upper panel: totalSTAT3/β-Tubulin. Lower panel: (pSTAT3/ β-Tubulin)/(totalSTAT3/ β-Tubulin). (**B**) Western Blot Analysis for STAT3 and pSTAT3 after miR-181a-5p mimic transfection in GBM42. Upper panel: totalSTAT3/β-Tubulin. Lower panel: (pSTAT3/ β-Tubulin)/(totalSTAT3/ β-Tubulin). Unpaired two-tailed students t-tests were applied to determine significance: * p < 0.05, ** p < 0.01, *** p < 0.001.


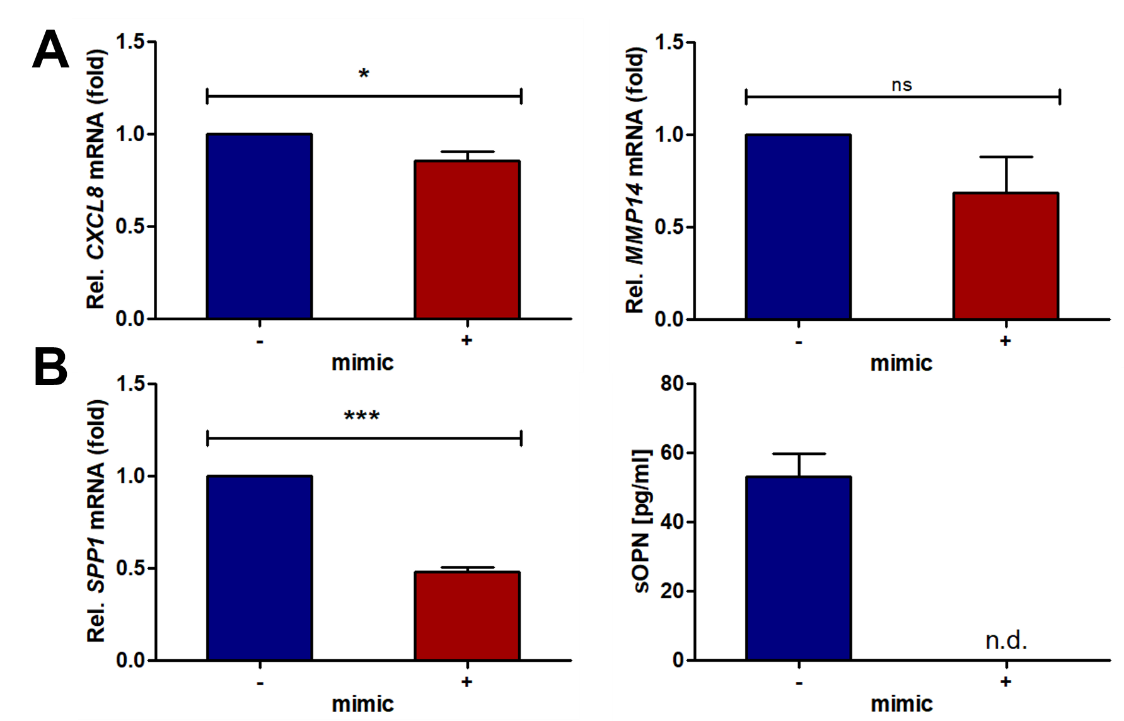


**Supplementary Figure 7.** *SPP1* and *CXCL8* are downregulated after miR-181a-5p mimic transfection. (**A**) RT-qPCR results of *CXCL8* (p=0.03) and *MMP14* (p=0.08) after transient miR-181a-5p mimic transfection. Results are derived from two independent experiments and shown in mean values +/- SD. (**B**) *SPP1* is downregulated (p=0.0006, n=2) after miR-181a-5p mimic transfection. Also, no soluble OPN could be detected via ELISA after miR-181a-5p mimic transfection (technical duplicates). Unpaired one-tailed students t-test was applied to determine significance: * p < 0.05, ** p < 0.01, *** p < 0.001.


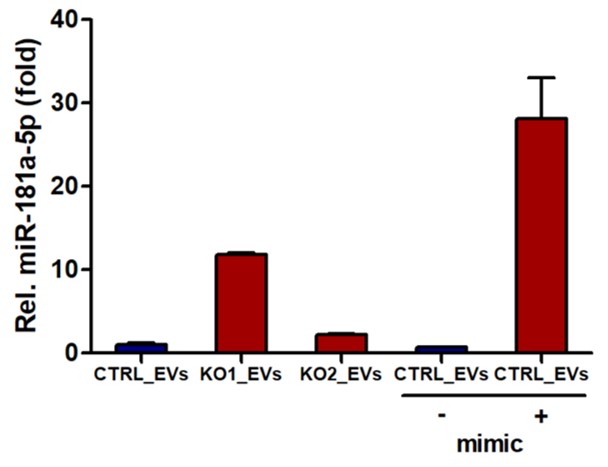


**Supplementary Figure 8.** MiR-181a-5p mimic transfected U87 cells release around 28-fold more miR-181a-5p in EVs than untransfected cells. The graph shows one RT-qPCR experiment used as a positive control in Figure 4D. Here, U87 cells were transfected with siRNA Ctrl or miR-181a-5p mimic before collection of supernatants and subsequent EV separation. MiRNA isolated from EVs show enrichment in mimic transfected samples, but also in EVs derived from U87_KO cells.


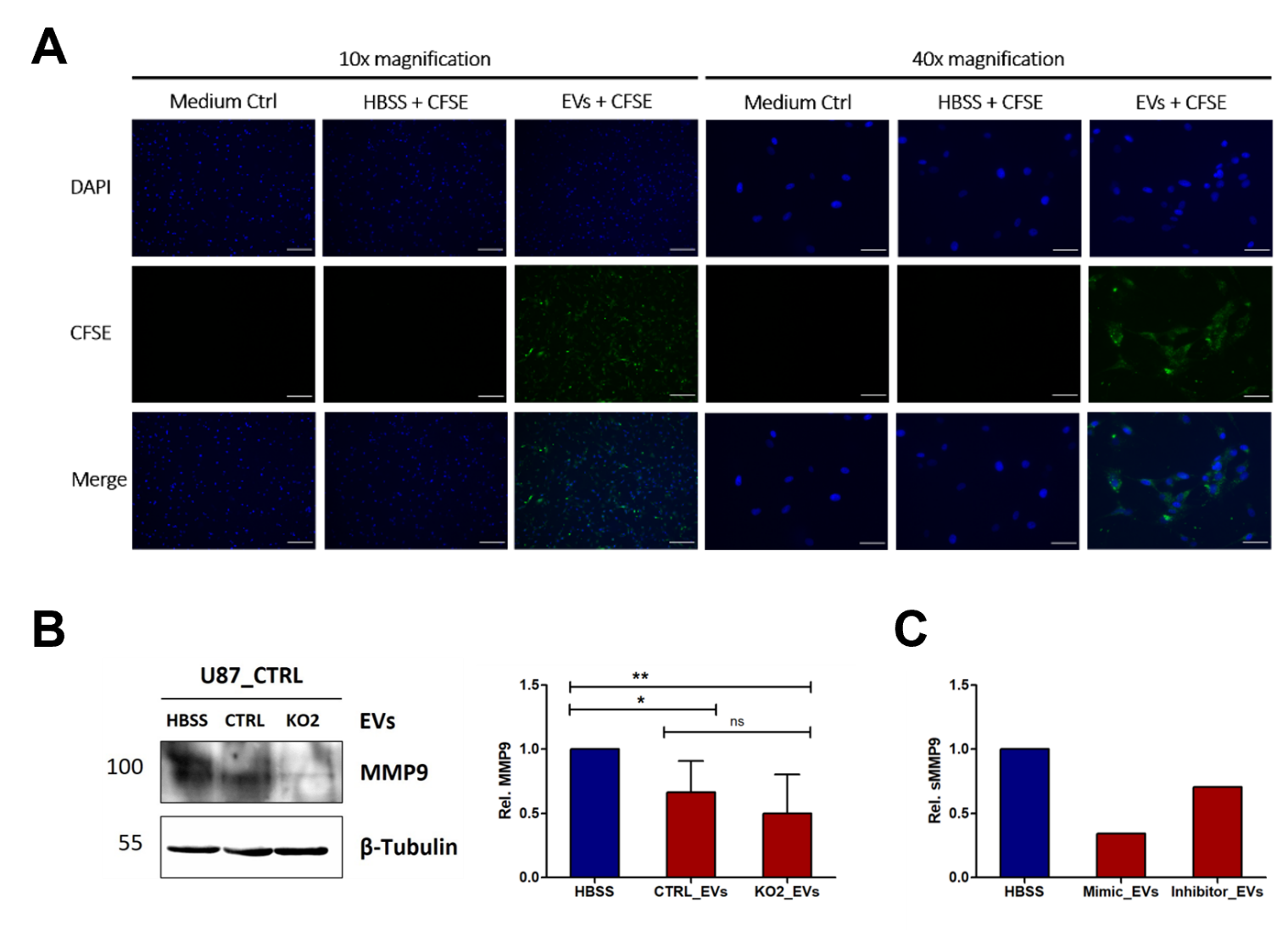


**Supplementary Figure 9. U87 derived EVs containing miR-181a-5p can contribute to effects across single cells. A** uptake of CFSE stained U87_KO derived EVs via U87_CTRL cells. U87_CTRL cells were incubated with HBSS only (left panel), HBSS + CFSE (middle panel) and CFSE stained EVs (right panel) for 5 hours. **B** U87_CTRL- and U87_KO-derived EVs encapsulating miR-181a-5p can inhibit MMP9 protein expression across single cells. One representative western blot is depicted on the left side, the graph on the right side describes the quantification of three independent experiments (CTRL_EVs) and five independent experiments in case of using KO2_derived EVs. Mean values +/- SD are shown. Unpaired students *t* test was applied to determine significance: * p < 0.05, ** p < 0.01 **C** The ELISA measurement of cellular supernatants indicates the decline of sMMP9 after treatment with mimic_EVs and the partial rescue by inhibitor_EVs. U87_CTRL cells were treated with EVs separated from cells transfected with miR-181a-5p mimic or miR-181a-5p inhibitor. Changes are depicted as fold change values to HBSS treated U87_CTRL cells.


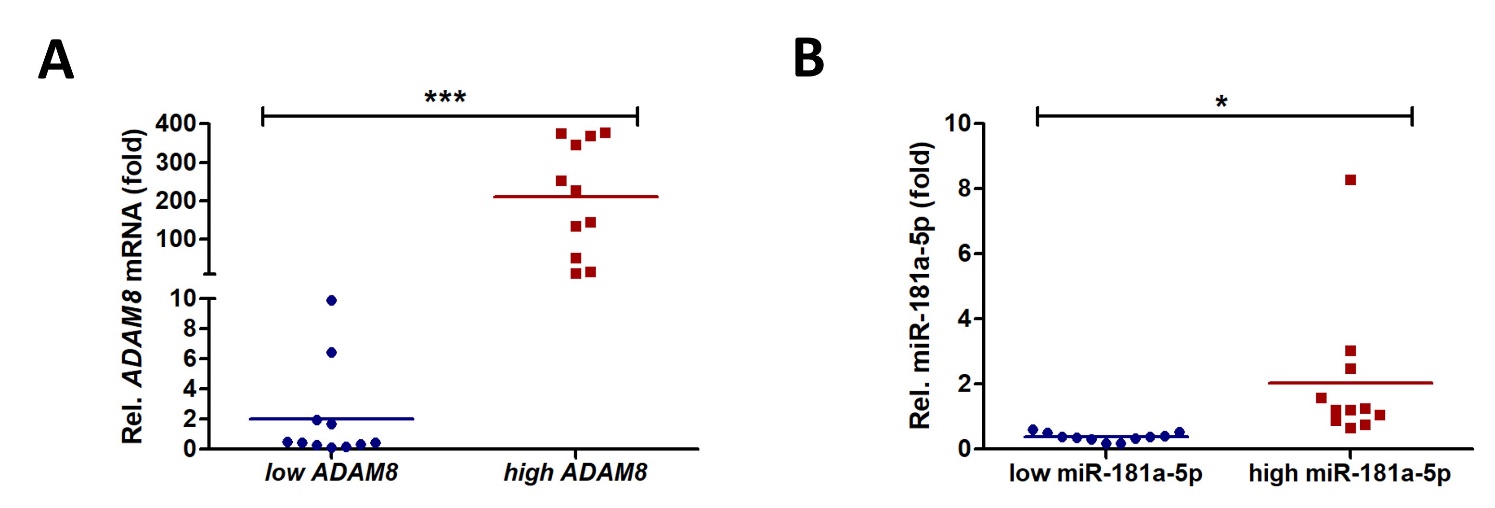


**Supplementary Figure 10.** Expression levels of *ADAM8* (A) and miR-181a-5p (B) in GBM tissue samples. The patient cohort (n=22) was divided into low/high *ADAM8* (A) or miR-181a-5p (B) expression using the median expression values. Unpaired two-tailed students t-tests were applied to determine significance: * p < 0.05, ** p < 0.01, *** p < 0.001.

## Supplementary Tables

| **Target** | **TargetScan** | **miRDB** | **Luciferase Assay validation** |
| --- | --- | --- | --- |
| **MEK1 (MAP2K1)** | Position 728-734 of MAP2K1 3' UTR 5'  ...UAAAUGGAAUUAUUUUGAAUGUC...  Position 286-292 of MAP2K1 3' UTR 5'   ...ACAGUGAAAUUUUGGUGAAUGUG... | Position 728-734 of MAP2K1 3' UTR  Position 286-292 of MAP2K1 3' UTR | He et al 2013 (1)  Wang P et al 2017 (2) |
| **ERK2 (MAPK1)** | Position 3685-3691 of MAPK1 3' UTR  5'  ...AUUUUAUUAAGAAUCUGAAUGUU...  Position 3842-3848 of MAPK1 3' UTR  5'  ...AAAAGAUGGAUUAUUUGAAUGUU... | Position 3685-3691 of MAPK1 3' UTR  Position 3842-3848 of MAPK1 3' UTR | He et al 2013 (1)  Wang P et al 2017 (2)  Huang et al. (2016) (3) |
| **CREB1** | Position 168-174 of CREB1 3' UTR  5'    ...UUUUGCAUUAAACUGUGAAUGUU...  Position 5049-5056 of CREB1 3' UTR  5'   ...GUUCUUAUUGAUUAGUGAAUGUA...  Position 7971-7977 of CREB1 3' UTR  5'  ...UUCAUAGUGCUGUUAUGAAUGUU... | Position 168-174 of CREB1 3' UTR  Position 5049-5056 of CREB1 3' UTR  Position 7971-7977 of CREB1 3' UTR | Fu et al. 2021 (4)  Liu et al 2013* (5) |
| **SPP1 (Osteopontin)** | Position 105-111 of SPP1 3' UTR  5' ...UCUCAGUUUAUUGGUUGAAUGUG...  Position 424-431 of SPP1 3' UTR  5'    ...AUAAAUCUUUUAUCUUGAAUGUA... | Position 105-111 of SPP1 3' UTR  Position 424-431 of SPP1 3' UTR | Marisetty et al 2020 (6) |
| **STAT3** | Position 277-283 of STAT3 3' UTR  5' ...AAAUAGAGAAAUGAGUGAAUGUG... | n.d. | n.d. |
| **MMP9** | n.d. | n.d. | n.d. |

**Supplementary Table 1.** Predicted targets of miR-181a-5p. As target prediction tools TargetScan and miRDB were utilized. A literature review was conducted to identify published Luciferase Assays (right column). Not detectable (n.d.) was used to state that either no target sequence was predictable or no publication with a corresponding luciferase assay was found. * This luciferase assay was conducted in rat-derived cells.

Mature Sequence of **hsa-miR-181a-5p**: 5’ *AACAUUCAACGCUGUCGGUGAGU* 3’

3’ *UGAGUGGCUGUCGCAACUUACAA 5’*

**References to Supplementary Table S1:**

1. He Q, Zhou X, Li S, Jin Y, Chen Z, Chen D, et al. MicroRNA-181a suppresses salivary adenoid cystic carcinoma metastasis by targeting MAPK-Snai2 pathway. Biochim Biophys Acta (2013) 1830(11):5258-66. doi: 10.1016/j.bbagen.2013.07.028

2. Wang P, Chen D, Ma H, Li Y. LncRNA SNHG12 contributes to multidrug resistance through activating the MAPK/Slug pathway by sponging miR-181a in non-small cell lung cancer. Oncotarget (2017) 8(48):84086-101. doi: 10.18632/oncotarget.20475

3. Huang X, Schwind S, Santhanam R, Eisfeld AK, Chiang CL, Lankenau M, et al. Targeting the RAS/MAPK pathway with miR-181a in acute myeloid leukemia. Oncotarget (2016) 7(37):59273-86. doi: 10.18632/oncotarget.11150

4. Fu Y, Xin Z, Ling Z, Xie H, Xiao T, Shen X, et al. A CREB1-miR-181a-5p loop regulates the pathophysiologic features of bone marrow stromal cells in fibrous dysplasia of bone. Mol Med (2021) 27(1):81. doi: 10.1186/s10020-021-00341-z

5. Liu Y, Zhao Z, Yang F, Gao Y, Song J, Wan Y. microRNA-181a is involved in insulin-like growth factor-1-mediated regulation of the transcription factor CREB1. J Neurochem (2013) 126(6):771-80. doi: 10.1111/jnc.12370

6. Marisetty A, Wei J, Kong LY, Ott M, Fang D, Sabbagh A, et al. MiR-181 Family Modulates Osteopontin in Glioblastoma Multiforme. Cancers (Basel) (2020) 12(12):3813. doi: 10.3390/cancers1212381

| **Tumor localization** | **Pathological findings** | **Choline** | **NAA** | **Cho/NAA** |
| --- | --- | --- | --- | --- |
| **L1** | Gray matter, slightly increased in the number of cells, access tissue | - | - | - |
| **L2** | Significantly increased number of cells in brain tissue, especially in white matter | - | - | - |
| **L3** | High cell density, tumor tissue | 14.5 | 19.2 | 0.76 |
| **L4** | High cell density, numerous vascular proliferation, local bleeding | 14.2 | 2.27 | 6.26 |

**Supplementary Table 2.** Detailed clinical information of the GBM patient analyzed for miR-181a-5p/*ADAM8*/*MMP9* expression in different tumor tissue areas. Results of the neuropathological histology and spectroscopy. Patient 25 is analyzed in Figure 5F-H. Cho: choline, NAA: N-acetyl-aspartate.
